# Inherited antithrombin deficiency caused by a mutation in the SERPINC1 gene: A case report

**DOI:** 10.1097/MD.0000000000031240

**Published:** 2022-11-04

**Authors:** Xinwei Hou, Kairu Zhang, Qian Wu, Mingyuan Zhang, Li Li, Hongwei Li

**Affiliations:** a Department of Oncology, Haihe Hospital, Tianjin University, Tianjin, China; b Tianjin Institute of Respiratory Diseases, Tianjin, China; c Department of Tuberculosis, Haihe Hospital, Tianjin University, Tianjin, China; d Department of Respiratory and Critical Care Medicine, Haihe Hospital, Tianjin University, Tianjin, China.

**Keywords:** gene duplication mutation, genetic testing, inherited antithrombin deficiency, SERPINC1 gene, thrombophilia

## Abstract

Inherited antithrombin deficiency (ATD) is a major cause of thrombotic deficiency. Genetic testing is of great value in the diagnosis of hereditary thrombophilia. Herein, we report a case of inherited ATD admitted to our hospital. We include the results of genealogy and discuss the significance of genetic testing in high-risk groups of hereditary thrombophilia.

**Patient concerns:**

A 16-year-old male patient presented with chest tightness, shortness of breath, wheezing, and intermittent fever (up to 39 °C) after strenuous exercise for 2 weeks. He also had a cough with white sputum with a small amount of bright red blood in the sputum and occasional back pain.

**Diagnoses:**

The blood tests showed that the patient’s antithrombin III concentration and activity were both significantly reduced to 41% and 43.2%, respectively. Enhanced chest computed tomography scans showed pulmonary infarction in the lower lobe of the right lung with multiple embolisms in the bilateral pulmonary arteries and branches. Lower vein angiography revealed a contrast-filling defect of the inferior vena cava and left common iliac vein. Thrombosis was considered as a differential diagnosis. His father and his uncle also had a history of thrombosis. The patient was diagnosed with inherited ATD. Further, peripheral venous blood samples of the family members were collected for whole-exome gene sequencing, and Sanger sequencing was used to verify the gene mutation site in the family. The patient and his father had a SERPINC1 gene duplication mutation: c.1315_1345dupCCTTTCCTGGTTTTTAAGAGAAGTTCCTC (NM000488.4).

**Interventions:**

An inferior vena cava filter was inserted to avoid thrombus shedding from the lower limbs. Urokinase was injected intermittently through the femoral vein cannula for thrombolysis. Heparin combined with warfarin anticoagulant therapy was sequentially administered. After reaching the international normalized ratio, heparin was discontinued, and oral warfarin anticoagulant therapy was continued. After discharge, the patient was switched to rivaroxaban as oral anticoagulation therapy.

**Outcomes:**

The patient’s clinical symptoms disappeared. reexamination showed that the thrombotic load was less than before, and the inferior vena cava filter was then removed.

**Lessons:**

By this report we highlight that gene detection and phenotypic analysis are important means to study inherited ATD.

## 1. Introduction

Thrombophilia refers to a group of diseases characterized by reduced anticoagulant and/or increased procoagulant substances in the blood due to hereditary or acquired factors. Patients with thrombophilia have a hypercoagulable state and high thromboembolic tendency,^[1]^ mainly manifesting as venous thromboembolism, such as deep vein thrombosis and pulmonary embolism (PE). These thromboembolisms also occur in some uncommon sites, such as splanchnic veins, cerebral veins, and retinal veins. There are several causes of thrombophilia,^[2]^ including anticoagulation protein deficiency,^[3]^ coagulation factor deficiency, fibrinolytic deficiency, metabolic deficiency, and underlying acquired diseases that cause hypercoagulability. Therefore, they are clinically divided into 2 types: inherited and acquired. Inherited antithrombin deficiency (ATD) is a major cause of thrombotic deficiency.^[4–6]^

Genetic testing is of great value in the diagnosis of hereditary thrombophilia. Clinical diagnosis is made based on medical history, family history, physical examination, laboratory tests, and diagnostic imaging data. Genetic testing and second-generation sequencing can help confirm the diagnosis. In this paper, we report a case of hereditary antithrombin deficiency in a patient admitted to our hospital. We include the results of genealogy and discuss the significance of genetic testing in high-risk groups of hereditary thrombophilia.

## 2. Materials and Methods

### 2.1. Ethical approval

The study was approved by the Ethics Review Committee of Haihe Clinical College of Tianjin Medical University, and all experiments were performed in accordance with approved guidelines. Informed consent was obtained from the patient’s parents.

### 2.2. Case history

A 16-year-old male patient was admitted to another institution for complaints of intermittent chest tightness and fever for 2 weeks. The patient had been previously healthy. His father had a history of PE and lower limb venous thrombosis. His uncle also had a history of venous thrombosis of the lower extremities. The patient developed chest tightness, shortness of breath, wheezing, and intermittent fever (up to 39 °C) after strenuous exercise, 2 weeks before admission to our hospital. The patient also had a cough with white sputum with a small amount of bright red blood in the sputum and occasional back pain. He visited the local hospital, and routine blood examination showed increased white blood cell count and neutrophil ratio (16.44 × 10^9^/L and 78.64%, respectively). C-reactive protein and D-dimer levels were increased (74.3 mg/L and 9295 ng/ml, respectively). Chest computed tomography (CT) showed a small, cloudy, high-density shadow in the right inferior lung lobe. After treatment with piperacillin sodium and sulbactam sodium for 1 week, the patient’s fever improved, but the chest tightness persisted. One day before admission to our hospital, lung perfusion scan showed decreased blood perfusion in both lungs, suggesting a high possibility of PE. The patient was then transferred to our hospital.

**Figure 1.**
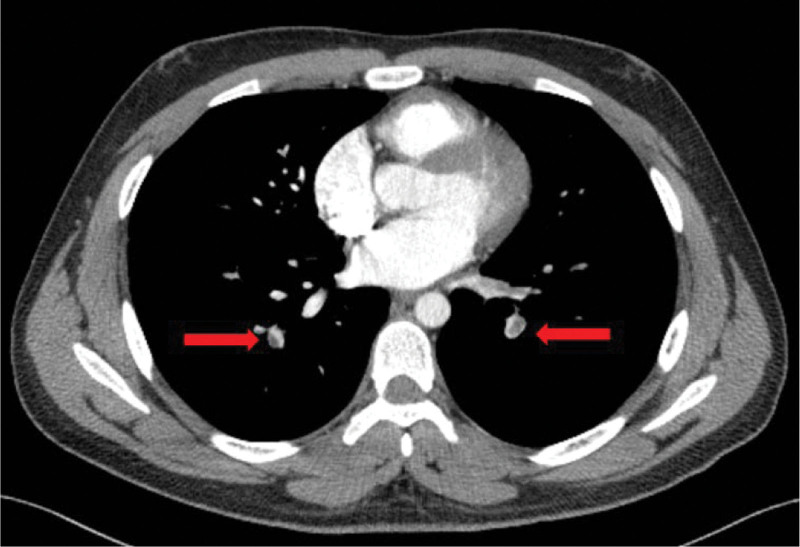
Enhanced chest computed tomography showing pulmonary infarction in the lower lobe of the right lung with multiple embolisms in bilateral pulmonary arteries and branches (red arrow).

**Figure 2.**
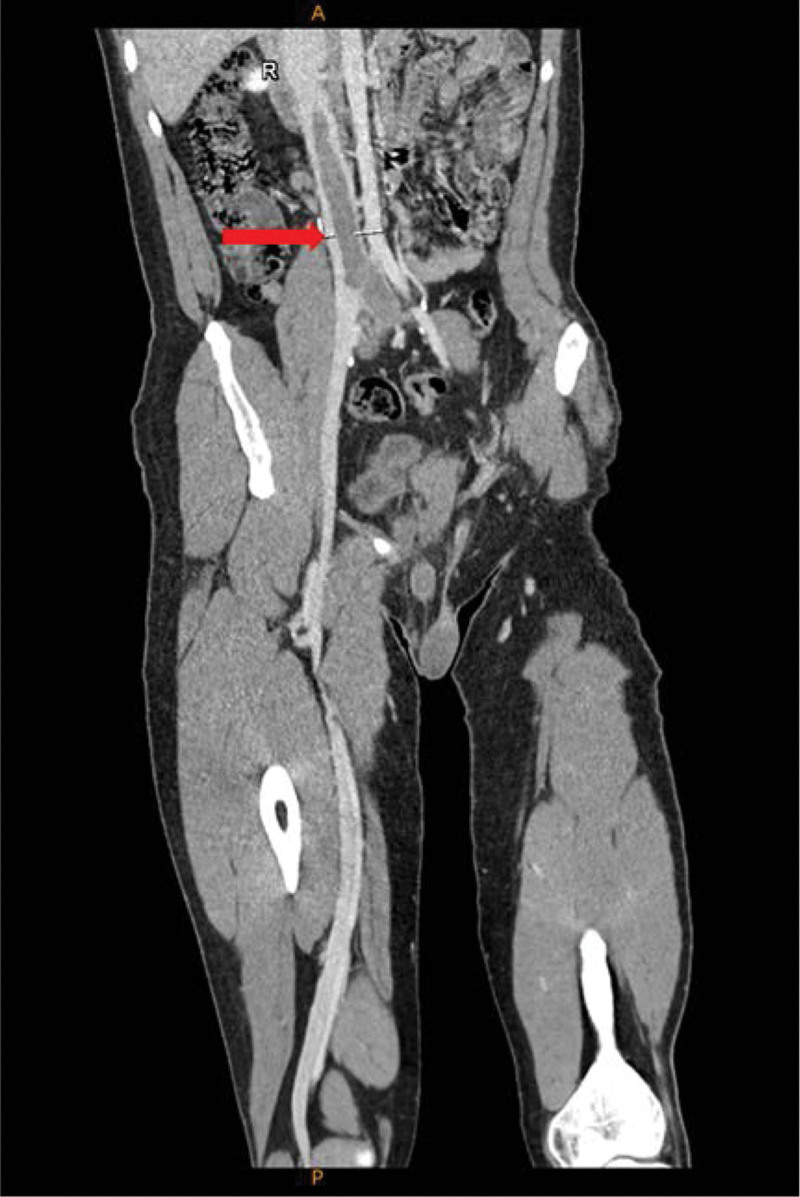
Lower vein angiography showing contrast filling defect of inferior vena cava and left common iliac vein (red arrow).

After admission, the examination showed that the patient’s antithrombin III concentration and activity were both significantly reduced to 41% (reference value: 75%–125%) and 43.2% (reference interval: 75%–125%), respectively. Enhanced chest CT scans showed pulmonary infarction in the lower lobe of the right lung with multiple embolisms in the bilateral pulmonary arteries and branches (Fig. 1). Lower vein angiography revealed a contrast-filling defect of the inferior vena cava and left common iliac vein. Thrombosis was considered as a differential diagnosis (Fig. 2). An inferior vena cava filter was inserted through the left internal jugular vein to avoid thrombus shedding from the lower limbs. During the operation, 200,000 U of urokinase was administered through the catheter for thrombolysis, the femoral vein cannula was retained after the operation, and 1.2 million U of urokinase was administered for thrombolysis, followed by heparin anticoagulation. Within 1 week, 200,000 U of urokinase was injected intermittently through the femoral vein cannula for thrombolysis. reexamination of enhanced chest CT showed decreased pulmonary thrombosis. However, lower vein angiography showed no significant reduction in inferior vena cava thrombosis or lower extremity deep venous thrombosis. Thrombolytic therapy with 800,000 U urokinase was administered intravenously via the inferior vena cava for 3 consecutive days. Heparin combined with warfarin anticoagulant therapy was sequentially administered. After reaching the international normalized ratio, heparin was discontinued, and oral warfarin anticoagulant therapy was continued. After discharge, the patient was switched to rivaroxaban as oral anticoagulation therapy. Six months after discharge, the patient was re-hospitalized for review. The examination showed that the thrombotic load was less than before, the inferior vena cava filter was removed, rivaroxaban oral anticoagulant therapy was continued after the operation, and a regular review was conducted.

### 2.3. Family gene sequencing and analysis

#### 2.3.1. Blood sample collection and gene sequencing.

Peripheral venous blood (5 mL) was collected from family members (the patient and the patient’s biological parents), and whole exome gene sequencing was performed by Novogene. The Agilent SureSelect Human All Exon V6 kit was used for sampling.

#### 2.3.2. Bioinformatics analysis.

Whole exome sequencing data were aligned with the hg19 human reference genome using BWA software (Wellcome Trust Sanger Institute, England). GATK software (Broad Institute, Cambridge, MA) was used to analyze insertion deletions and single-nucleotide polymorphism sites. Annovar software (Children’s Hospital of Philadephia, Philadelphia, PA) and 1000 genomes, dbSNP, and OMIM databases were used for annotation. Polyphen2 software (Brigham & Women's Hospital, Boston, MA) and SIFT software (Craig Venter Institute, Rockville, MD) were used to predict the protein function.

#### 2.3.3. Sanger sequencing

According to the whole-exome sequencing results, Sanger sequencing was used to verify the proband and his father. Polymerase chain reaction (PCR) was used to amplify the genes of the proband, proband’s father, and healthy controls. Primers were designed to amplify a mutant SERPINC1 gene c.1315_1345dupCCTTTCCTGGTTTTTATAAGAGAAGTTCCTC (NM000488.4). The upstream primer was 5′-TGGGTTACCTGAATGGAACT-3′ and the downstream primer was 5′-ACACAAGGGTTGGCTACTCT-3′. The PCR reaction conditions were as follows: pre-degeneration at 94 °C for 2 minute, 94 °C for 30 seconds, 56 °C for 30 seconds, 72 °C for 35 seconds, 72 °C for 2 minute, 35 cycles. The PCR products were detected using 1.5% agarose gel electrophoresis. The unpurified amplification products were sent to Tianjin Anshengda Biotechnology Company for Sanger sequencing. These sequencing results were compared with reference sequences in NCBI GenBank using Chromas software to determine the mutation sites. The online software Human Splicing Finder (http://www.umd.be/HSF/) was used for gene splice site prediction.

## 3. Results

**Figure 3.**
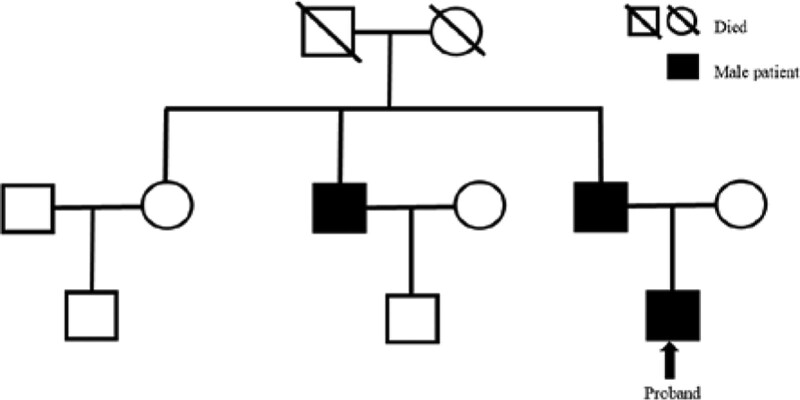
The family tree of the proband.

In this family, the proband, proband’s father, and proband’s uncle all had a history of venous thrombosis, and there was a familial genetic predisposition for the condition. (Fig. 3).

**Figure 4.**
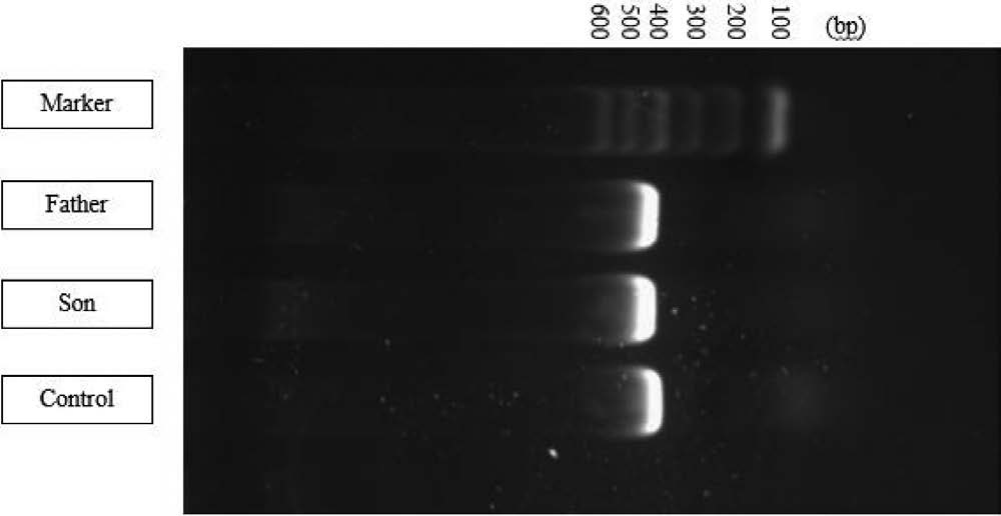
PCR analyses of SERPINC1 amplification. PCR = polymerase chain reaction.

**Figure 5.**
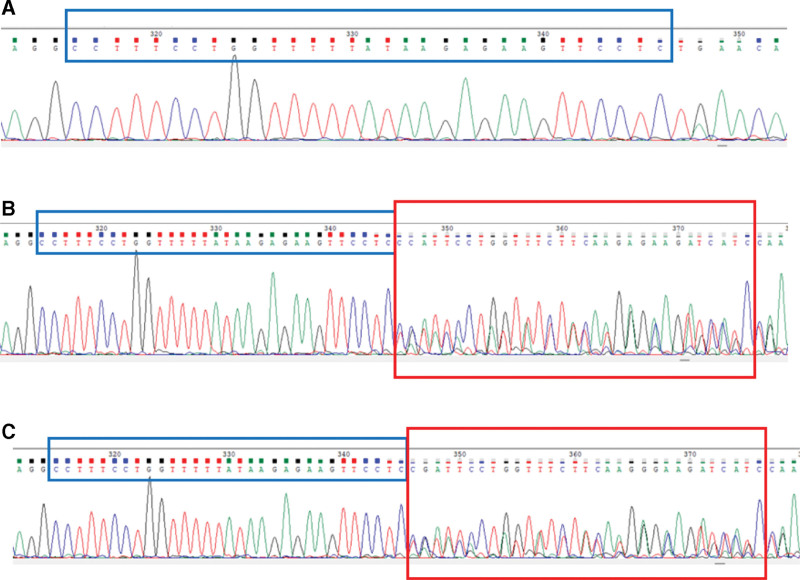
The peak figure of the son and his father (A – control, B – father, C – son).

By collecting the peripheral venous blood of the proband and his parents and performing whole-genome and Sanger sequencing verification, it was confirmed that both the proband and his father had a SERPINC1 gene duplication mutation: c.1315_1345dupCCTTTCCTGGTTTTTAAGAGAAGTTCCTC (NM000488.4). This variant was not detected in the proband’s mother. Visualization of PCR products showed that the fluorescent bands of the proband and the father of the proband were brighter and wider than those of the control, and the band position was between 400 bp and 500 bp. There was no significant difference between the proband and the father (Fig. 4). The sequencing results were further compared with the reference sequence in GenBank using the Chromas software, and the mutation site was determined to be c.1315_1345dupCCTTTCCTGGTTTTTAAGAGAAGTTCCTC (Table 1 and Fig. 5). This mutation was identified for the first time by searching the NCBI database (https://www.ncbi.nlm.nih.gov/).

**Table 1:** The SERPINC1 sequences of the proband and his father.

Group	Sequence
Genebank	CCGCTGTTGTGATTGCT GGCCGTTCGCTAAACCCCAA CAGGGTGACTTTCAAGGCCAACA GG**CCTTTCCTGGTTTTTA** **TAAGAGAAGTTCCTC**TGAACA CTATTATCTTCATGGGCAGAG
Control	CCGCTGTTGTGATTGCTGGCC GTTCGCTAAACCCCAACA GGGTGACTTTCAAGGCCAAC AGG**CCTTTCCTGGTTTTTA** **TAAGAGAAGTTCCTC**TGAAC ACTATTATCTTCATGGGCAGAG
Father	CCGCTGTTGTGATTGCTGG CCGTTCGCTAAACCC CAACAGGGTGACTTTCAAGG CCAACAGG**CCTTTCCTGGTTTT** **TATAAGAGAAGTTCCTC** **CCTTTCCTGGTTTTTATAAG** **AGAAGTTCCTC**TGAACACTATT ATCTTCATGGGCAGAG
Proband	CCGCTGTTGTGATTGCTGGCC GTTCGCTAAACCCCAACAGGG TGACTTTCAAGGCCAACAGG**C** **CTTTCCTGGTTTTTATAAGA** **GAAGTTCCTCCCTTTCCTG** **GTTTTTATAAGAGAAGTTCCTC** TGAACACTATTATC TTCATGGGCAGAG

## 4. Discussion

Antithrombin (AT), a serine protease inhibitor, is the most important anticoagulant in the blood circulation system, accounting for 50% to 70% of the total anticoagulant activity in plasma.^[7]^ It is the most known effective endogenous anticoagulant and is key to maintaining the balance between coagulation and anticoagulation in vivo and preventing thromboembolism.^[8,9]^ There are 4 subtypes of antithrombin, of which only antithrombin III (and possibly thrombin I) is clinically significant. For that reason, antithrombin is often referred to as antithrombin III. The molecular structure of the antithrombin consists of 3 fragments, 9 helices, and 1 reaction center ring, which contains the “decoy sequence” of the target protease. Heparin can regulate antithrombin activity by accelerating the interaction between antithrombin and the target protease. Therefore, heparin and its derivatives are the main drugs used for the treatment of thromboembolism.

Antithrombin deficiency (ATD) is a rare autosomal dominant hereditary disease. It has been reported that up to 80% of hereditary antithrombin deficiency are caused by mutation of the antithrombin gene (SERPINC1).^[10,11]^ The antithrombin gene is located on autosomal chromosome 1q23–25 and consists of 6 exons to generate a construct of 13.4 kb. According to the ratio of antithrombin antigen content to antithrombin activity, hereditary AT deficiency can be divided into 2 types: type I, quantitative deficiency, which manifests as reduced antithrombin content and thus its activity, and type II, qualitative deficiency, which only manifests as decreased antithrombin activity.^[8]^ With the application of gene sequencing technology, hundreds of SERPINC1 gene mutations have been found to cause antithrombin deficiency.^[10]^ Among them, type I antithrombin deficiency is commonly associated with missense mutations, frameshift deletions, nonsense mutations, insertion and deletion of small fragments (<30 bp), and deletion of large fragments or whole gene segments.^[12]^ Type II antithrombin deficiency is divided into 3 subtypes (IIa, IIb, and IIc), and the mutation types are missense mutations without a hotspot mutation region.

Thrombotic events caused by ATD usually occur before the age of 20, and most patients present with symptoms between the ages of 4 and 50 years. Thrombosis may be caused by trauma, surgery, or other factors, mostly affecting the venous system; however, a few cases of arterial thrombosis have been reported. Common sites of thrombosis include the leg, mesenteric, and superficial periumbilical veins.^[13]^

In our case, an adolescent male child admitted to our hospital presented with intermittent fever, chest pain and tightness, blood in the sputum, and a history of exercise before onset.

Enhanced chest CT revealed a PE, and lower vein angiography showed multiple thromboses in the inferior vena cava, bilateral common iliac vein, bilateral external iliac vein, bilateral internal iliac vein, bilateral femoral vein, bilateral popliteal vein, and bilateral posterior tibial vein. Blood tests showed that antithrombin and antithrombin activities were significantly lower than the lower limit of the normal value. The patient’s father and uncle had a history of venous thrombosis. Based on the patient history, family history, laboratory tests, and gene sequencing results, hereditary antithrombin deficiency type I was diagnosed. After active thrombolytic and anticoagulant therapy, the patient’s symptoms improved, and he was discharged.

Genomics, genetic and phenotypic analyses of families with inherited ATD have become an important means to study this type of disease. A large retrospective cohort study of 540 heterozygous carriers with SERPINC1 mutations found that the risk of thrombotic events could be evaluated by identifying the mutation types through detection of the SERPINC1 gene.^[6]^ SERPINC1 genetic testing can also identify the etiology of AT deficiency-related arterial thrombosis in ischemic stroke patients.^[14]^ In addition, genetic testing can also assess the risk of recurrence in patients and make asymptomatic diagnosis of family mutation gene carriers,^[15,16]^ which has important guiding significance for the selection of individualized treatment plans.

## 5. Conclusions

In conclusion, gene detection and phenotypic analysis are important means to study inherited ATD. For young patients with multiple venous thromboembolisms, family history should be carefully investigated, and genetic analysis and family investigations should be carried out for those with a clear diagnosis, which will help to identify pathogenic mutations, assess the risk of thrombosis and recurrence, select appropriate anticoagulant drugs, improve disease prognosis, and provide the basis for thromboprophylaxis interventions in asymptomatic carriers.

## Author contributions

**Conceptualization:** Li Li, Hongwei Li.

**Data curation:** Xinwei Hou, Kairu Zhang, Qian Wu, Mingyuan Zhang, Li Li, Hongwei Li.

**Formal analysis:** Xinwei Hou, Kairu Zhang, Qian Wu, Mingyuan Zhang, Li Li, Hongwei Li.

**Funding acquisition:** Hongwei Li.

**Investigation:** Xinwei Hou, Kairu Zhang, Qian Wu, Mingyuan Zhang, Li Li, Hongwei Li.

**Methodology:** Xinwei Hou, Qian Wu.

**Project administration:** Hongwei Li.

**Resources:** Hongwei Li.

**Software:** Xinwei Hou, Hongwei Li.

**Supervision:** Hongwei Li.

**Writing – original draft:** Xinwei Hou, Hongwei Li.

**Writing – review & editing:** Xinwei Hou, Hongwei Li.
